# Health workforce needs of small medical specialties: findings from rheumatology in Germany

**DOI:** 10.1093/eurpub/ckac129.323

**Published:** 2022-10-25

**Authors:** E Kuhlmann, K Hoeper, T Witte, D Ernst, A Dopfer-Jablonka

**Affiliations:** 1 Clinic for Rheumatology and Immunology, Medizinische Hochschule Hannover, Hannover, Germany; 2 Regionales Kooperatives Rheumazentrum Nieders, Hannover, Germany

## Abstract

**Background:**

Small medical specialties may be more vulnerable to workforce shortage and the COVID-19 pandemic and this may directly impact in the provision of care for chronically-ill patients. This study aims to explore health workforce development and new needs, using rheumatology in Germany as a case study.

**Methods:**

An explorative multi-methods approach was applied, combining health labour market assessment of rheumatology physicians (public statistics 2000-2019) and a questionnaire-based online survey conducted in early 2021 (n = 101 respondents; rheumatology physicians and residents). Main selected topics: work hours, workload, mental health issues, discrimination and sexual harassment experiences, impact of COVID-19. Descriptive statistical analysis was performed and qualitative content analysis for free-text information.

**Results:**

Health labour market analysis showed that the numbers of rheumatologists increased markedly between 2000 and 2019 in the groups aged +50 years, but only 9% in younger groups under 50 years; since 2010 the group 40-50 years showed decreases. In 2019, the absolute number of rheumatologists working in healthcare after retirement-age exceeded those aged 40 and under. Survey data revealed a strong mismatch between actual and desired work hours for women and men. 81% rated their workload as high or very high; every sixth rheumatologist has suffered from stress or burnout syndromes at least once in the past. Experiences of gender discrimination and sexual harassment/violence were frequently reported, mostly by women. COVID-19 was an amplifier of stress with major stressors being digitalisation and increased demand for communication and patient education.

**Conclusions:**

Decreasing health workforce capacities in German rheumatology combine with negative perceptions of work and workplace conditions, threatening both retention and service delivery.

**Key messages:**

• Small medical specialties, like rheumatology, face severe shortage that threaten healthcare for chronically-ill patients and need greater attention.

• COVID-19 has reinforced rheumatologists’ workload and stressors, thus worsing mental health and retention.

